# Clinical evaluation of Bladder CARE, a new epigenetic test for bladder cancer detection in urine samples

**DOI:** 10.1186/s13148-021-01029-1

**Published:** 2021-04-21

**Authors:** Paolo Piatti, Yap Ching Chew, Michiko Suwoto, Taikun Yamada, Benjamin Jara, Xi-Yu Jia, Wei Guo, Saum Ghodoussipour, Siamak Daneshmand, Hamed Ahmadi, Jeffrey Rice, Jeffrey Bhasin, Faith Holloway, Yvonne Tsai, Yoshitomo Chihara, Gangning Liang

**Affiliations:** 1 Zymo Research Corp, Irvine, CA USA; 2 Pangea Laboratory LLC, Costa Mesa, CA USA; 3 Department of Urology, Keck School of Medicine, University of Southern California, Los Angeles, CA USA

**Keywords:** Bladder cancer, DNA methylation, At-home sample collection, Non-invasive testing, Urine

## Abstract

**Background:**

Bladder cancer (BC) is the 5th most common cancer in the USA. Non-muscle invasive bladder cancer represents about 70% of all cases and has generally a favorable outcome. However, recurrence rates as high as 60 to 70% and progression rates of 10 to 20% necessitate intensive surveillance with cystoscopy. The invasiveness and high cost of cystoscopy poses significant burden on BC patients as well as on the healthcare system. In this study we test the feasibility of a simple, sensitive, and non-invasive detection of BC using Bladder CARE test in urine samples.

**Results:**

Urine from 136 healthy and 77 BC subjects was collected using the at-home Bladder CARE Urine Collection Kit and analyzed with Bladder CARE test. The test measures the methylation level of three BC-specific biomarkers and two internal controls using methylation-sensitive restriction enzymes coupled with qPCR. Bladder CARE showed an overall sensitivity of 93.5%, a specificity of 92.6%, and a PPV and NPV of 87.8% and 96.2%, respectively. Bladder CARE has an LOD as low as 0.046%, which equates to detecting 1 cancer cell for every 2,200 cells analyzed. We also provided evidence that bisulfite-free methods to assess DNA methylation, like Bladder CARE, are advantageous compared to conventional methods that rely on bisulfite conversion of the DNA.

**Conclusion:**

Highly sensitive detection of BC in urine samples is possible using Bladder CARE. The low LOD of the test and the measurement of epigenetic biomarkers make Bladder CARE a good candidate for the early detection of BC and possibly for the routine screening and surveillance of BC patients. Bladder CARE and the at-home urine sample collection system have the potential to (1) reduce unnecessary invasive testing for BC (2) reduce the burden of surveillance on patients and on the healthcare system, (3) improve the detection of early stage BC, and (4) allow physicians to streamline the monitoring of patients.

**Supplementary Information:**

The online version contains supplementary material available at 10.1186/s13148-021-01029-1.

## Background

Bladder cancer (BC) is the 5th most common cancer in the USA [1] with 81,400 new cases and 17,980 deaths estimated for 2020 and an incidence rate of 2.4% [2]. BC occurs more frequently in Caucasians compared to other ethnicities, and it is three to four times more frequent in men than in women, making it the 4th most common cancer in men in the USA and the 8th most common cause of cancer death [1–3]. Tobacco smoking is the primary risk factor for BC, and it is estimated to contribute to the development of up to 50% of all bladder tumors. Other well-documented risk factors include chronic urinary tract infections, occupational exposure to carcinogens (e.g., aromatic amines, polycyclic aromatic hydrocarbons), chronic exposure to arsenic, pelvic radiation, and genetic predisposition [4–9]. The most common histology is urothelial carcinoma; approximately 70% of all cases are non-muscle invasive bladder cancer (NMIBC), while the remaining 30% are represented by muscle invasive bladder cancer (MIBC) [10, 11]. NMIBC has a generally favorable outcome after transurethral resection of bladder tumor (TURBT; 80% 10-year survival). However, recurrence rates as high as 60 to 70% and progression rates of 10 to 20% necessitate intensive surveillance for NMIBC. Current guidelines recommend lifelong surveillance with cystoscopy [12–14]. While cystoscopy is the gold standard for the detection of bladder tumors, it is invasive, costly, and poses significant burden on the patient and on the healthcare system. The costs of treatment and surveillance make BC the most expensive cancer to manage [15].

In addition to cytology, a number of tests for the non-invasive detection of BC have been developed including Nuclear Matrixprotein-22 (NMP22) [16], the ImmunoCyt assay (Scimedx, Denville, NJ, USA) [17], Bladder Tumor-associated Antigen (BTA) [18], the UroVysion (Abbott Molecular Inc., Des Plaines, IL, USA) [19], Cxbladder [20], UroMark [21], and EpiCheck [22]. The adoption of some of these tests in the clinical practice for the detection of BC is becoming a reality, and it is currently being extensively evaluated. A summary of the performance characteristics of different non-invasive BC tests in comparison with cytology and cystoscopy is shown in Table 1 [21, 23–28].

**Table 1:** Performance characteristics of different non-invasive BC tests

Test name	Principle	Sensitivity%	Specificity%
Bladder CARE	Multiplex MSRE-qPCR	94*	93*
BTA *stat*	Immunoassay	61 [23]	78 [23]
Cxbladder	RT-qPCR	91 [24]	Not reported
EpiCheck	Multiplex MSRE-qPCR	63 [25, 26]	86 [25, 26]
ImmunoCyt	Immunocytochemical assay	62 [23]	79 [23]
NMP22 BladderChek	Immunoassay	58 [23]	85 [23]
UroMark	Targeted bisulfite sequencing	98 [21]	97 [21]
UroVysion	Fluorescence in situ hybridization (FISH)	72 [27]	83 [27]
Cytology		48 [23]	86 [23]
Cystoscopy (blue light)		92 [28]	48 [28]
Cystoscopy (white light)		81 [28]	49 [28]

Test type and performance characteristics of several non-invasive BC tests are summarized and compared to cytology and cystoscopy techniques

^*^Performance of Bladder CARE is based on the results reported in this manuscript

DNA methylation is an epigenetic mark that is often altered in many human diseases including cancer [29, 30], and it is believed that alterations in DNA methylation are early events in tumorigenesis [11, 31]. DNA methylation, therefore, promises to be a good biomarker for early tumor detection, especially when multiple methylation biomarkers are combined in a multiplex panel [11]. In patients affected by BC, tumor DNA and cells are released in the urine, allowing for the non-invasive detection of cancer-specific methylation biomarkers from urine specimens for both diagnostic and monitoring purposes as described in pioneering works [11, 32–35]. DNA methylation is commonly analyzed by treating DNA with sodium bisulfite and then detected using next-generation sequencing and PCR-based approaches [36, 37]. DNA conversion with sodium bisulfite, however, introduces DNA strand breaks and results in the degradation of up to 84–96% of the input DNA [38], with a consequent reduction of the number of DNA molecules that can be effectively analyzed. This problem is particularly pronounced in samples that contain low or already highly fragmented DNA (e.g., urine DNA and cell-free DNA).

In this study we evaluated the feasibility of Bladder CARE (Pangea Laboratory LLC, CA, USA) in the detection of BC in urine samples. Bladder CARE is a newly developed test that uses methylation-sensitive restriction enzymes to analyze the methylation level of three BC-specific methylation biomarkers (TRNA-Cys, SIM2, and NKX1-1). These loci are hypermethylated in both NMIBC and MIBC and were discovered and validated in previous studies [39]. In the current study we aim to determine the clinical performance of Bladder CARE on a cohort of healthy controls and patients with BC, evaluate the advantages of bisulfite-free methods for the assessment of DNA methylation, and test the feasibility of at-home sample collection.

## Results

### Study population cohort demographics

The study population included a total of 213 subjects. In total, 77 urine samples were collected from individuals affected by BC (cancer cohort) and 136 from healthy subjects (control cohort). The characteristics of the study population are represented in Table 2.

**Table 2:** Clinicopathological characteristics of the cohort included in the study (*n* = 213)

Characteristic	Control cohort	Cancer cohort
	*n* = 136	*n* = 77
*Age (years)*		
Mean (range)	50.4 (23–88)	66.4 (47–86)
*Sex, no. (%)*		
Male	72 (52.9)	65 (84.4)
Female	64 (47.1)	12 (15.6)
*Ethnicity, no. (%)*		
Caucasian	100 (73.5)	74 (96.1)
Asian	22 (16.2)	2 (2.6)
African-American	3 (2.2)	0 (0)
Hispanic	1 (0.7)	1 (1.3)
Other	0 (0)	0 (0)
Not available	10 (7.4)	0 (0)
*Tumor type, no. (%)*		
NMIBC, TCC	n.a	66 (85.7)
MIBC, TCC	n.a	10 (13.0)
AC	n.a	1 (1.3)
SCC	n.a	0 (0)
*Tumor grade, no. (%)*		
Low grade	n.a	10 (13.0)
High grade	n.a	51 (66.2)
Not Available	n.a	16 (20.8)

*NMIBC* non-muscle invasive bladder cancer, *MIBC* muscle invasive bladder cancer, *TCC* transitional cell carcinoma, *AC* adenocarcinoma, *SCC* squamous cell carcinoma, *n.a.* not applicable

The cancer cohort had an average age of 66.4 years, was 84.4% male, and 96.1% Caucasian. In total, 13% of the BC samples were collected from subjects affected by low-grade NMIBC tumor, while 66.2% from individuals affected by high-grade urothelial carcinoma (41 NMIBC and 10 MIBC). Grading information was not available for the remaining 20.8% of the samples.

The control cohort had an average age of 50.4 years and was 52.9% male. The cohort was 73.5% Caucasian, 16.2% Asian, 2.2% African-American, and 0.7% Hispanic. No ethnicity information was available for the remaining 7.4% of the cohort.

### Bladder CARE correctly classifies the cohorts included in the study population

Bladder CARE results between the cancer and the control cohorts were compared. Bladder CARE results are expressed as the Bladder CARE Index (BCI). BCI values are calculated by integrating the methylation level of the three BC biomarkers and the two internal controls in a proprietary algorithm developed by Pangea Laboratory [39, 40].

**Fig. 1:**
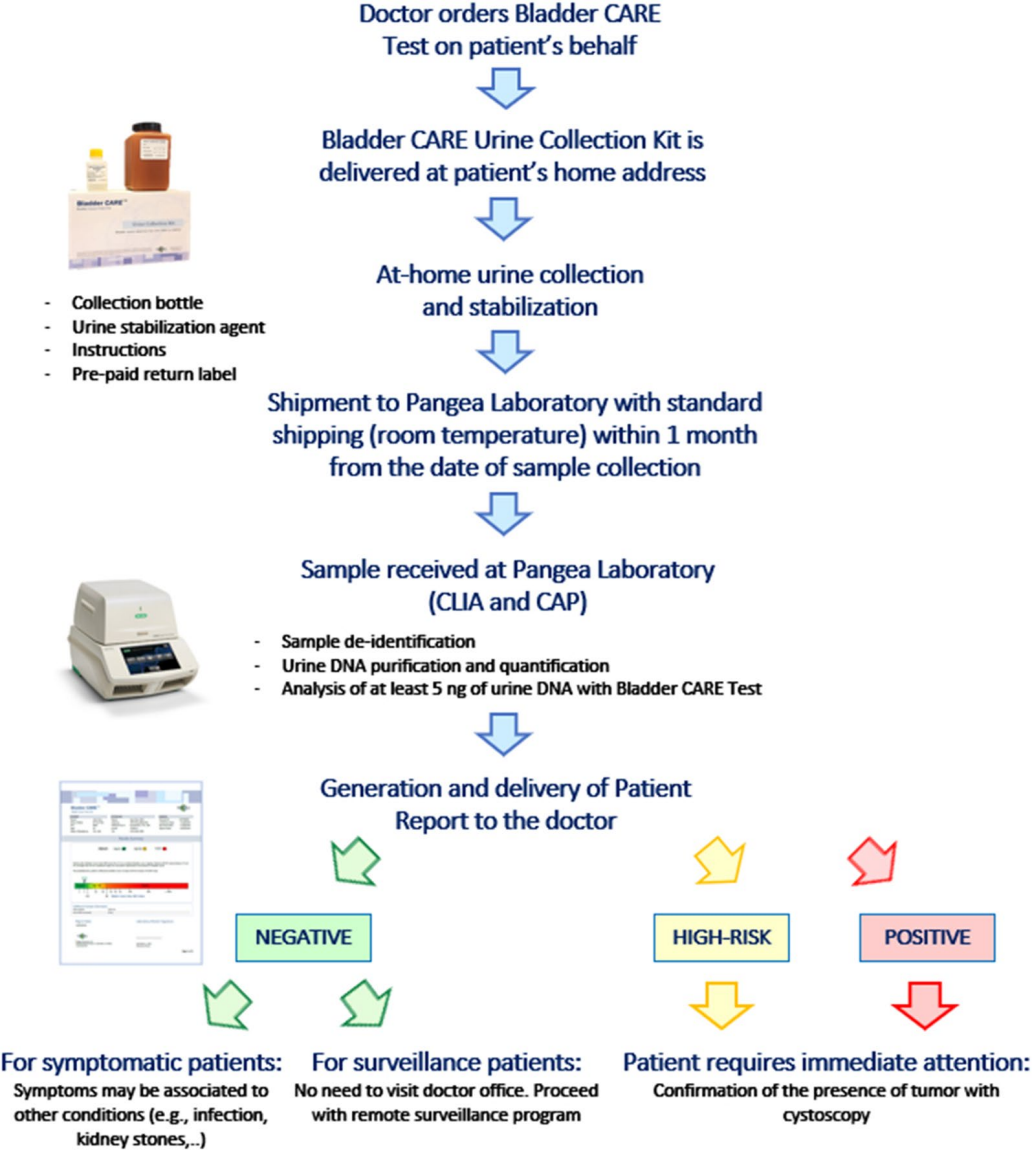
Bladder CARE workflow. After the test has been ordered, Bladder CARE Urine Collection Kit is directly mailed to the patient’s home address. A stabilization agent (Urine Preservation Reagent; Pangea Laboratory, PNG100-1-35) is added to the urine right after the sample collection in order to preserve the urine DNA integrity for up to one month at room temperature [42]. Upon return mailing, urine samples are processed at Pangea Laboratory and at least 5 ng of urine DNA is analyzed with Bladder CARE test. A sample report is sent to the doctor. Patients can be classified as Negative, High-Risk, and Positive by Bladder CARE

Based on BCI values, samples are categorized as ‘Negative,’ ‘High-Risk,’ and ‘Positive.’ Specifically, samples with BCI < 2.5 are considered Negative for the presence of BC, while samples with BCI between 2.5 and 5, and > 5 are classified as High-Risk and Positive, respectively ([40], Fig. 1).

**Fig. 2:**
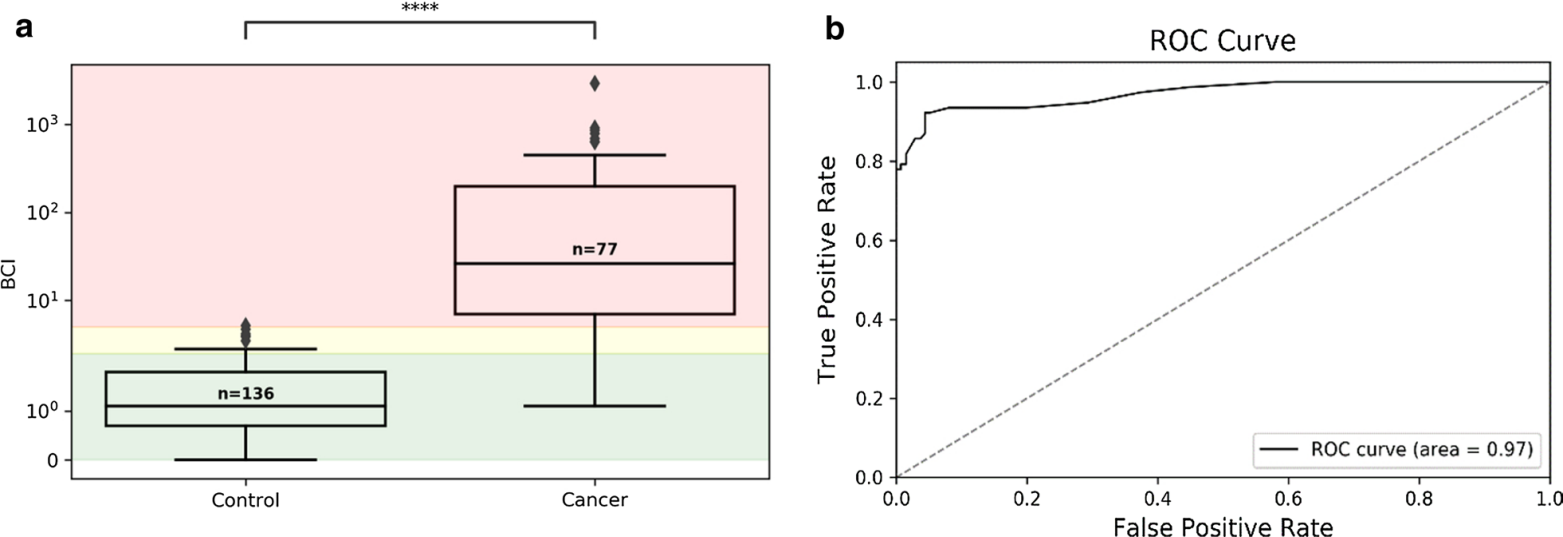
Bladder CARE test correctly classifies control and cancer cohorts. **a** Distribution box plots of BCI values (*Y*-axis) represented on a symmetrical log scale (*Y*-axis is linear between 0 and 2; *Y*-axis > 2 is illustrated on a base 10 logarithmic scale). The size of each cohort is indicated above each median. Statistical significance (calculated using Student’s two-tailed *t* test) between cohorts is indicated on the top of the figure. Interquartile range (the range of samples from the 25th to 75th percentile) is represented by the box, and the cohort median value is represented by the horizontal line within the box. Outliers (values outside the minimum and maximum represented by the whiskers, equaling the 25th or 75th percentile + 1.5 * the interquartile range) are indicated by black diamonds. Bladder CARE-negative samples (BCI < 2.5) are delimited by the green area, while High-Risk and Positive samples (BCI between 2.5 and 5, and > 5) are delimited by the yellow and red areas, respectively. **b** Receiver operating characteristic (ROC) curve using the BCI value for classification. The area under the curve (AUC) of the ROC curve is a measure of classification performance, plotting the difference in true-positive rate (sensitivity) and false-positive rate (1—specificity) as the classification decision boundary is changed (what value is required to classify as positive), where 0.5 = no discrimination and 1 = perfect discrimination. Classification thresholds were taken at a 0.25 BCI interval, with a resulting AUC of 0.971

We observed significantly higher BCI values for BC patients compared to BC-free volunteers (average BCI value for the cancer and control cohorts was 170.1 and 1.3, respectively; *p* = 6.2 × 10^–7^; Fig. 2a). Calculated 95% CIs for the cancer and the control cohorts were 84.39 to 255.61 and 1.173 to 1.487, respectively. As shown in Fig. 2b the ROC curve AUC was 0.971 (1 being perfect discrimination, 0.5 being no better than chance).

### Bladder CARE has high sensitivity, specificity, and predictive values

To evaluate the sensitivity of Bladder CARE we analyzed 77 urine samples collected from the cancer cohort. Five cancer subjects resulted Negative (false-negative calls), while the other 72 were classified as High-Risk (*n* = 12) and Positive (*n* = 60) by Bladder CARE (Table 4A). Samples resulting High-Risk and Positive were grouped together as positive for the calculation of the overall test sensitivity, which resulted in 93.5% (94.1% and 90.0% for high- and low-grade tumors, respectively. Tables 3, 4A, B). We did not observe a significant difference in the average BCI values between low-grade and high-grade cancer sub-cohorts (156.5 and 207.6, respectively; *p* = 0.7310).

**Table 3:** Bladder CARE test performance

Total cohort (*n* = 213)	
True positive	72
True negative	126
False positive	10
False negative	5
Sensitivity%	93.5
Specificity%	92.6
PPV%	87.8
NPV%	96.2

Performance of Bladder CARE test calculated from the analysis of the control (*n* = 136) and cancer (*N* = 77) cohorts included in this study

**Table 4:** Sensitivity of Bladder CARE for the cancer cohort

(**A**)			
**Cancer cohort** **(*n* = 77)**			
True positive (Positive + High-Risk)		72 (60 + 12)	
False negative		5	
Total sensitivity %		93.5	

(**B**)			
**Tumor grade**	**True positive**	**False negative**	**Sensitivity %**
Low grade	9	1	90.0
High grade	48	3	94.1
Grade not specified	15	1	93.8

(A) Sensitivity of Bladder CARE calculated for the cancer cohort (*n* = 77), and (B) for the cancer cohort classified based on tumor grade

To gain information about the specificity of Bladder CARE, urine samples of 136 healthy individuals were analyzed. Bladder CARE has an overall specificity of 92.6% (Table 3). Ten false-positive cases (9 High-Risk and 1 Positive) with an average BCI of 3.6 (range 2.7–5.2) were detected within the control cohort (Table 5A). When we classified the control population based on sex, age, or ethnicity (Table 5B), we did not observe significant differences in BCI; however, we noticed that the majority of the false-positive cases were found among male individuals (7 out of 10), between 50 and 80 years old (9 out of 10) and Caucasian (9 out of 10).

**Table 5:** Sensitivity of Bladder CARE

** (A)**		
**Control cohort (*n*** **= 136)**		
True negative	126	
False positive	10	
Total specificity %	92.6	

**(B)**		
	**True negative**	**False positive**
**Ethnicity**		
Caucasian	91	9
Asian	22	0
African-American	3	0
Hispanic	1	0
Other	n.a	n.a
Not available	9	1
**Sex**		
Male	65	7
Female	61	3
**Age**		
20–29	19	0
30–39	30	1
40–49	12	0
50–49	23	3
60–69	24	3
70–79	16	3
80–89	2	0

(A) Specificity of Bladder CARE calculated for the control cohort (*n* = 136). (B) Distribution of the false-positive cases based on the cohort classification accordingly to ethnicity, sex, and age; n.a., data not available

Positive and negative predictive values (PPV and NPV) for Bladder CARE were 87.8% and 96.2%, respectively (Table 3).

### Bladder CARE test results can estimate cancer probability

Patients are classified as Negative, High-Risk, or Positive by Bladder CARE. This classification already helps doctors to identify which patients require immediate attention; however, a precise estimation of cancer probability may add another layer of information that can help doctor’s decision, especially for patients with High-Risk results.

**Fig. 3:**
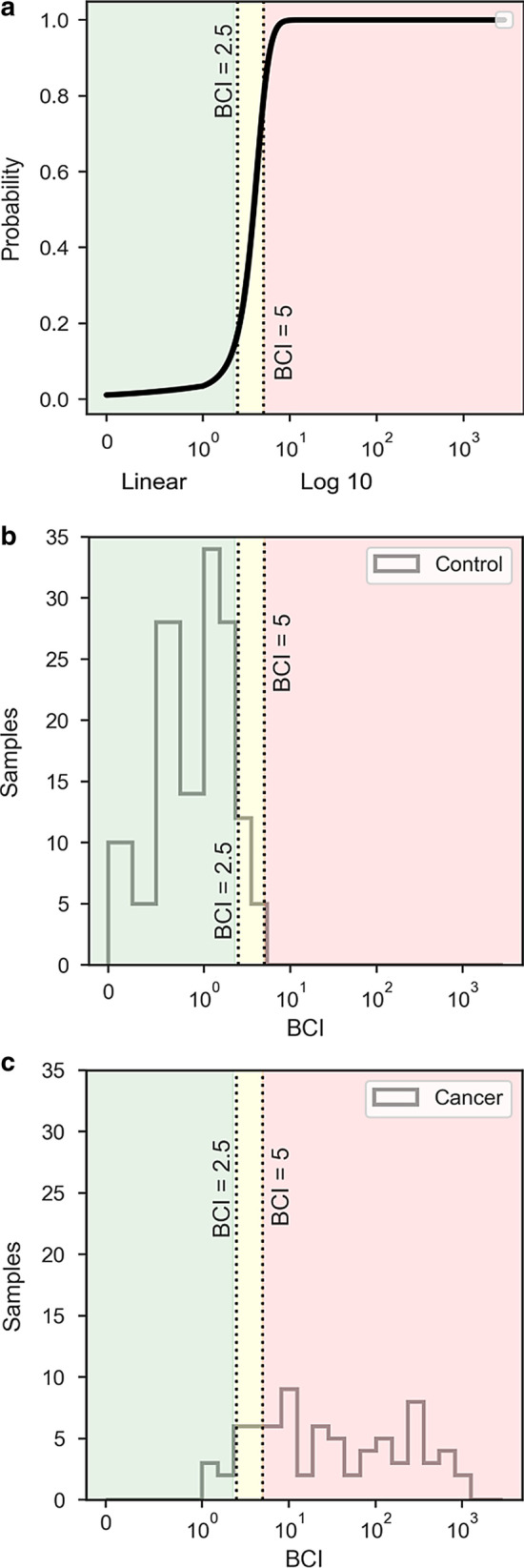
Correlation between BCI and probability of being affected by BC. **a** Probability of bladder cancer (*Y*-axis) based on BCI values (*X*-axis) as determined by the logistic regression model. **b**, **c** Sample frequencies by their BCI value as histogram plots for the control and cancer cohorts. Cohort BCI values are divided into 23 bins, divided evenly on a base 10 logarithmic scale from 1 to the max BCI value for 19 bins, with four additional bins representing BCI values from 0 to 1 at 0.25 increments. BCI values (*X*-axis) are represented using a symmetrical log scale (BCI values between 0 and 1 are represented on a linear scale, while values > 2 are illustrated on a base 10 logarithmic scale). Bladder CARE classifications are delimited by shading, with Negative (BCI < 2.5) as green, and High-Risk (BCI 2.5–5) and Positive classifications (BCI > 5) delimited by the yellow and red areas, respectively

To provide a continuous estimated probability of cancer, a logistic regression model was generated utilizing the BCI values and clinical information available for the cancer and control cohorts (Fig. 3). The resulting model had an *F*_1_-Score of 0.9 (a measure of binary classification accuracy on a 0–1 scale, with 1 representing perfect precision and recall) and a receiver operating characteristics (ROC) area under the curve (AUC) score of 0.98 (Additional file 1: Figure S1). The AUC score is a measure of model performance when considering model output as a classifier (on a 0.5–1 scale, with AUC = 1 signifying perfect discrimination).

A plot estimated probability of cancer versus BCI value is shown in Fig. 3a, compared to histogram plots highlighting the distribution of the cancer and control cohorts by BCI value (Fig. 3b, c). The model indicates a low estimated cancer probability for BCI values below 2.5 and highlights a sharp increase in cancer probability into the High-Risk classification with an estimated cancer probability of 80% at a BCI value of 5 (Fig. 3a).

Figure 3b, c shows that the majority (92.6%) of the control subjects were classified as such, while most of cancer subjects were classified as Positive and High-Risk (77.9% and 15.6%, respectively).

### Bisulfite-free methods maximize qPCR signal

Bisulfite treatments to study DNA methylation cause significant sample degradation [31, 38]. In contrast, bisulfite-free approaches like Bladder CARE are believed to minimize the loss of DNA [31], potentially contributing to the overall test sensitivity.

**Fig. 4:**
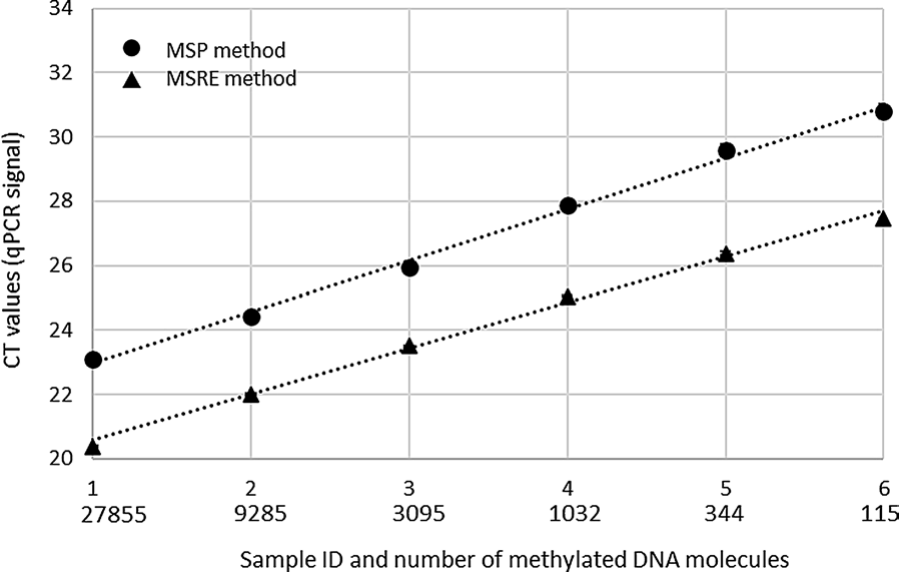
Comparison between signal generated from MSRE-qPCR and MS-qPCR. The methylation level of the CpG island of the human MGMT gene was analyzed in a set of 6 spike-in samples containing different amount of artificially methylated and unmethylated DNA. Signals originated from MSRE-qPCR and MS-qPCR are marked by black rectangles and black circles, respectively. Cycle threshold (CT) values are indicated on the *Y*-axis. Sample IDs and calculated methylated DNA copy number (considering that one DNA molecule weighs 3.59 pg [43]) originally present in the samples before restriction digestion or bisulfite conversion is indicated on the *X*-axis. Each sample was tested using 100 ng of spike-in DNA in three technical replicates

To validate this hypothesis, we analyzed 6 spike-in samples containing different amounts of artificially methylated and untreated blood DNA with methylation-sensitive restriction enzymes (MSRE) qPCR (a bisulfite-free method comparable to Bladder CARE) and with methylation-specific (MS) qPCR method (a common bisulfite-based method). The genomic region selected for this experiment was the CpG island of the human MGMT gene, which is unmethylated in blood DNA samples as shown in Additional file 2: Table S1. Besides the treatment (enzyme digestion or bisulfite conversion) the other variables (primer binding sites, qPCR conditions, and input DNA used) were kept constant between the two experiments. As shown in Fig. 4, despite starting from the same amount of DNA, we found that samples analyzed with MSRE-qPCR amplify on average 2.83 PCR cycles earlier compared to the same samples analyzed with MS-qPCR. This corresponds to a substantial gain in qPCR signal (~ 86%) for samples digested with methylation-sensitive restriction enzymes compared to bisulfite-treated samples.

### Bladder CARE can detect 1 cancer cell for every 2,200 normal cells

**Fig. 5:**
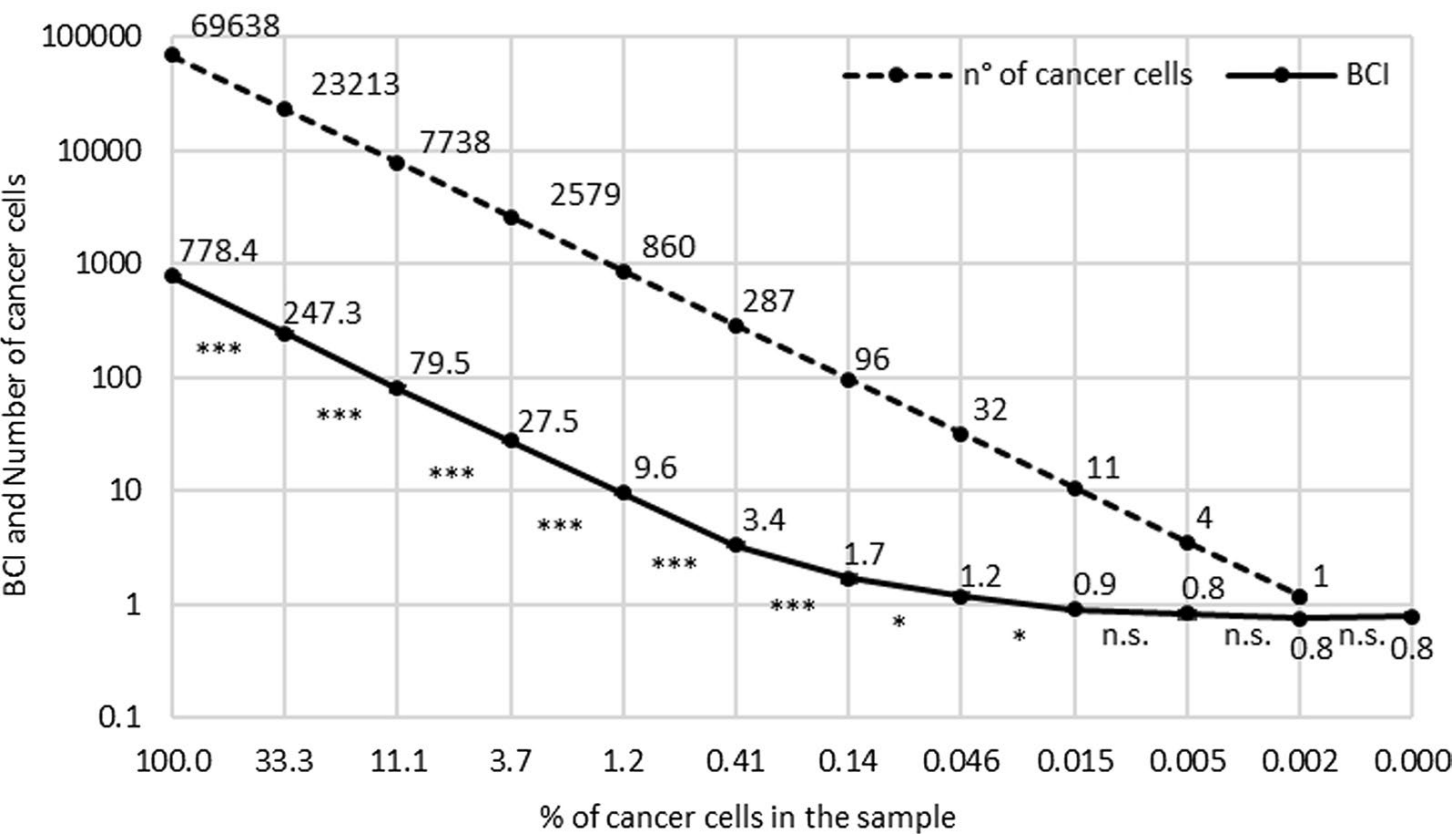
Determination of Bladder CARE linearity and LOD. Bladder CARE linearity and LOD: comparison between the number of cancer cells (LD583 cancer cell line [41]) originally present in the 12 spike-in samples (dashed line and numbers above it) and the correspondent Bladder CARE Index (BCI) value (solid line and numbers above it). Each sample was tested using 500 ng of spike-in DNA in three technical replicates. Significant differences in BCI between two consecutive samples are indicated by asterisks below the solid line. n.s. = not significant. Although barely visible, error bars for technical replicates are reported for each spike-in sample. Percentage of cancer cells in each spike-in sample (500 ng, correspondent to approximately 70,000 cells considering that one DNA molecule weighs 3.59 pg [43]) is indicated in the *X*-axis. BCI value and number of cancer cells are represented in the *Y*-axis (logarithmic scale)

To gain information about the limit of detection (LOD) of Bladder CARE, we submitted to Pangea Laboratory a set of 12 artificial samples (Additional file 2: Table S2) containing different proportions of LD583 BC cell line DNA [41] and blood DNA isolated from a healthy donor. Blood DNA was used as “background DNA” since Bladder CARE markers are not methylated in blood [39]. Results in Fig. 5 indicate that BCI values decrease linearly with a decrease in the number of cancer cells present in the samples until a concentration of 0.14%. BCI differences between consecutive samples remain significant until sample 8, indicating that Bladder CARE significantly detects the presence of cancer cells at a minimum concentration (LOD) of 0.046% (*p* = 0.031; Fig. 5 and Additional file 2: Table S2). This is the equivalent of detecting 1 cancer cell in a sample containing 2200 normal cells. Below this limit BCI values do not significantly deviate from the control sample 12 (0% of cancer cell content; Fig. 5 and Additional file 2: Table S2). We must point out, however, that Bladder CARE classifies as High-Risk and Positive in samples with a BCI greater than 2.5. This number corresponds to an LOD between 0.14 and 0.41% (Fig. 5, Additional file 2: Table S2). Therefore, while the LOD of Bladder CARE can be as low as 0.046%, the LOD of the test in the current setup is between 0.14 and 0.41%.

## Discussion

The measurement of epigenetic tumor markers in urine specimens represents a non-invasive alternative to detect the presence of BC. Alterations in DNA methylation are believed to be early events in tumorigenesis [14]; therefore, epigenetic tumor biomarkers promise to be good candidates for the early cancer detection of BC.

In this study we tested the performance of a new non-invasive and bisulfite-free epigenetic test for BC detection, Bladder CARE, and the feasibility of at-home urine sample collection (Fig. 1). We used urine specimens collected from 77 BC patients and 136 healthy subjects (Table 2). We found that Bladder CARE effectively detects the presence of BC (both, high- and low-grade tumors; Table 4B) with an overall sensitivity of 93.5%, a specificity of 92.6%, and a PPV and NPV of 87.8% and 96.2%, respectively (Table 3).

Within the control cohort we found 10 false-positive cases (Table 5A), potentially indicating that Bladder CARE may overestimate the presence of BC. Interestingly, the majority of false-positive cases were found among the most at-risk populations for BC: males (7 out of 10 false-positive cases) [3], Caucasians (9 out of 10 false-positive cases) [1, 2], and those between 50 and 80 years old (9 out of 10 false-positive cases) [1, 2] (Table 5B). It is possible that Bladder CARE detects early epigenetic alterations associated with pre-cancerous lesions which may not be yet associated with symptoms or a proper tumor formation. This may explain the apparent false-positive cases detected by Bladder CARE. However, one limitation of this study is that no clinical and follow-up information was available for the control cohort (“healthy” status was based on self-report); therefore, these suppositions are purely speculative, and more data need to be generated in the future order to better understand the reason for false-positive cases.

Within the cancer cohort we found 5 false-negative cases (Table 4A). A closer look at the data did not show any apparent correlation between false-negative cases and tumor type, grade, urine DNA yield, or presence of blood and leukocytes in the urine (Additional file 2: Table S3). Previously conducted interference experiments for urinary tract infections and hematuria reveal that these conditions do not interfere with Bladder CARE test results (data not shown). A possible explanation for false-negative cases is that the concentration of cancer cells in these samples may have been below Bladder CARE’s LOD. Additional studies are required to fully elucidate this aspect.

Bladder CARE effectively detected the presence of both, high- and low-grade BC tumors with a sensitivity of 94.1% and 90.0%, respectively (Table 4B). The high sensitivity of the test for both BC grades may be linked to the nature of epigenetic cancer biomarkers. Methylation status may become altered early during tumorigenesis and independently from the tumor stage or grade [39]. Although low-grade tumors rarely develop into aggressive and metastatic disease, their sensitive detection is still relevant from a diagnostic point of view since this type of tumor needs to be treated and patients followed to monitor for recurrence of the tumor. We must point out, however, that the number of low-grade BC samples included in this study is limited and future studies are necessary to confirm the highly sensitive detection of these type of tumors with Bladder CARE.

Urine samples for Bladder CARE are collected and stabilized at-home using the Bladder CARE Urine Collection Kit and can be mailed at ambient temperature [42]. None of the patients included in this study reported difficulties during remote sample collection, and all the samples received contain enough DNA to perform the Bladder CARE test. The remote sample collection and stabilization represents an important element of novelty in the field. It is advantageous for both the patient (especially for those that do not have easy access to the healthcare provider) and the physician since it can reduce otherwise unnecessary visits to the doctor office while still allowing close monitoring of the patients. Remote sample collection may also significantly lower the costs of surveillance and monitoring of BC patients.

We found that Bladder CARE has an LOD as low as 1 cancer cell for each 2,200 cells tested, and that the results are linear with the concentration of cancer cells present in the sample (Fig. 5, Additional file 2: Table S2). The low LOD may have important implications for early cancer detection. In addition, tumor signal can be monitored with Bladder CARE after TURBT or during treatments such as neoadjuvant chemotherapy in order to obtain information about the completeness of the resection procedure or the tumor response to treatments. This may allow urologists to intervene selectively and in a timely manner in cases where an increase of BCI is observed. We need to point out, however, that the LOD has been calculated in an artificial setting, using bladder cancer cell line DNA spiked in a background of genomic DNA; therefore, differences between the LOD reported in the manuscript and the true clinical LOD may exist.

We also provided evidence that bisulfite-free methods like Bladder CARE allow for maximal cancer signal in qPCR (Fig. 4) by preserving the integrity of cancer-specific methylated DNA regions. This is an advantage compared to methods that rely on bisulfite conversion of the DNA and which are known to cause significant DNA degradation [31, 38]. Any loss of DNA (especially in samples with low and fragmented DNA like urine DNA and cell-free DNA) may have significant negative effects on the test result and may increase false-negative cases.

Finally, we also generated a model for estimating probability of cancer based on BCI results (Fig. 3a–c, Additional file 1: Figure S1). Knowing the probability of cancer in addition to Bladder CARE patient classification (Negative, High-Risk, and Positive) may further help a physician’s decision making, especially for those patients with High-Risk Bladder CARE results.

## Conclusion

Our study indicates that Bladder CARE is a good candidate for the simple, sensitive, and non-invasive detection of BC. While cystoscopy remains the gold standard to confirm the presence of BC and the first intervention procedure to remove BC, Bladder CARE has the potential to reduce unnecessary cystoscopies and visits to the doctor office, allowing at the same time a close surveillance of BC patients. We envision that Bladder CARE, along with the Urine Collection Kit, could be used as a first-line test for the detection of BC and for the surveillance of those with a history of BC.

## Methods

### Study population

The study cohort consisted of patients with histologically confirmed urothelial carcinoma and healthy donors. The cancer cohort included specimens that were purchased from Geneticist Inc. (Glendale, CA, USA), and samples collected at the Department of Urology, USC Norris Comprehensive Cancer Center (Los Angeles, CA, USA), in accordance with an institutional review board approved protocol. The presence of BC, as well as the tumor type and grade, was confirmed by cystoscopy and histology from resections or biopsies. No exclusion criteria were applied for BC patients. Samples from healthy donors were collected from consenting volunteers over 21 years old. “Healthy” status was based on self-reporting and defined as no history of any type of tumor. No follow-up information was collected for both cohorts.

### Urine sample collection, stabilization, and shipment

Urine samples were collected and stabilized using the Bladder CARE Urine Collection Kit (Pangea Laboratory). No specific guidelines were established for the timing of urine sample collection. Samples purchased from Geneticist Inc. (Glendale, CA, USA) were analyzed with Multistix 10SG Urinalysis Test Strip (Siemens) prior to stabilization. Stabilized urine samples can be kept at room temperature for up to one month without DNA degradation or loss [42] and were mailed at ambient temperature to Pangea Laboratory for Bladder CARE analysis. A detailed description of the Bladder CARE workflow is presented in Fig. 1.

### Clinical procedures and Bladder CARE test

Stabilized urine was processed and analyzed at Pangea Laboratory. After isolation using *Quick*-DNA™ Urine Kit (Zymo Research, D3061), urine DNA samples were quantified with Femto™ Human DNA Quantification Kit (Zymo Research, E2005). As low as 5 ng of urine DNA was analyzed in duplicate with Bladder CARE.

Briefly, the test measures the methylation level of three BC-specific biomarkers and two internal control loci (the last informing about the input DNA amount used in the test and the efficiency of the digestion step for each sample tested) using methylation-sensitive restriction enzymes coupled with qPCR [39]. Positive and negative Bladder CARE control samples were also analyzed in parallel with clinical samples in order to confirm the validity of the test. A detailed description of the method is reported elsewhere [39, 40].

### Calculation of Bladder CARE test results (Bladder CARE Index—BCI)

Bladder CARE results are expressed as the Bladder CARE Index (BCI). BCI values are calculated by integrating the methylation level of the three BC biomarkers and the two internal controls in a proprietary algorithm developed by Pangea Laboratory [39, 40].

Based on BCI values, samples are categorized as ‘Negative,’ ‘High-Risk,’ and ‘Positive.’ Specifically, samples with BCI < 2.5 are considered Negative for the presence of BC, while samples with BCI between 2.5 and 5, and > 5 are classified as High-Risk and Positive, respectively ([40], Fig. 1).

### Statistical analyses

Results from a previous pilot study (57 healthy subjects and 51 BC patients; unpublished) were used to define each group’s mean BCI values with 95% CIs for the control and the cancer cohorts (control mean of 1.3 with a 95% CI of 0.994 to 1.61 and cancer mean of 213.7 with a 95% CI of 86.7 to 341). We conducted a power analysis using the n_2t_unequal() function from the dvmisc package in R, which allows group-specific variances (the cancer group has a much higher variance than control). Using the group-specific variances from the pilot data, an alpha threshold of 0.05, and a difference of means of 200 BCI units, we established that we would need 45 samples per group to achieve 80% power in a comparison of control and cancer groups. In our study we included 77 BC patients and 136 healthy subjects. Using the power_2t_unequal() function we calculated that a sample size of 77 per group would result in 96.2% power at alpha of 0.05 to detect a difference in means of 200 BCI units.

Sensitivity, specificity, PPV and NPV of Bladder CARE were calculated based on the number of true-positive, true-negative, false-positive, and false-negative cases. All the samples classified as Negative by Bladder CARE (BCI < 2.5) were considered negative in our study, while all the samples having a BCI > 2.5 (High-Risk and Positive Bladder CARE results) were considered positive.

The statistical significance of differences in BCI values was determined using Student’s two-tailed *t* tests. Box plots of the cohorts by BCI values and the receiver operating characteristic (ROC) curve for the BCI classification (Fig. 2) were generated in python 3.7.2 using numpy 1.16.2 and pandas 0.24.2 packages for data processing, custom code to generate the true-positive rates and false-positive rates for the ROC curve sliding threshold, scikit-learn 0.20.3 to calculate the ROC area under the curve (AUC), and seaborn 0.9.0 with matplotlib 3.0.2 for visualization.

To provide an estimated probability of BC occurrence based on the BCI (Fig. 3, Additional file 1: Figure S1), a logistic regression model was developed utilizing BCI values and their corresponding clinical sample information (cystoscopy results and self-report). Logistic regression modeling was evaluated using a stratified *k*-fold cross-validation approach, where the dataset was divided into five similarly sized subsets containing a ratio of outcomes representative of the total dataset. Model selection and generation was then repeated five times (training with four of the subsets and testing with one), rotating the subset to be tested. Diagnostics measurements were generated for each fold, with the resulting model being chosen where the measurements most closely matched the mean of the cross-validation diagnostics. Calculations were performed in python 3.7.2 using the numpy 1.16.2 and pandas 0.24.2 packages for data processing, statsmodels 0.9.0 for model generation, scikit-learn 0.20.3 for additional validation, and seaborn 0.9.0 with matplotlib 3.0.2 for visualization.

### Comparison between MSRE-qPCR and MS-qPCR

Six standard DNA samples (800 ng each) were generated by combining different proportions of untreated and artificially methylated blood DNA (produced using M.SssI CpG Methylase; Zymo Research, E2011). Specifically, samples 1 to 6 contained 100%, 33.3%, 11.1%, 3.7%, 1.23%, and 0.41%, of artificially methylated DNA. In total, 400 ng of each sample was bisulfite-treated using EZ DNA Methylation-Lightning Kit (Zymo Research, D5031), while the remaining 400 ng was digested using 10U of the methylation-sensitive restriction enzyme HpaII (New England BioLabs, R0171S) and purified with DNA Clean and Concentrator-5 (Zymo Research, D4013). Samples were then amplified by qPCR (CFX96 Touch qPCR System, Bio-Rad) using primers designed for bisulfite-converted methylated DNA (MS-qPCR) and genomic untreated DNA (MSRE-qPCR). Primers (sequences available upon request) have similar efficiencies and were designed on the same region of the CpG island of the human MGMT gene, which is known to be unmethylated in blood samples collected from healthy donors (Additional file 2: Table S1). Both amplicons have similar length and were amplified in triplicates in 20 µl of reaction containing ZymoTaq qPCR Premix (Zymo Research, E2055), 0.4 µM of each primer, and the equivalent of 100 ng of input DNA prior to bisulfite conversion or digestion. The program used to generate both amplicons has an initial 10-min denaturation step at 95 °C followed by 40 cycles of denaturation at 97 °C for 20 s and annealing/extension at 58 °C for one minute. Data were analyzed using Bio-Rad CFX Maestro Software (Bio-Rad).

Methylation of the human MGMT target region in untreated and artificially methylated blood DNA was determined by MSRE-qPCR according to the protocol described above. Results are represented in Additional file 2: Table S1.

### LOD and linearity of Bladder CARE

A set of 12 spike-in samples containing different amounts of LD583 (bladder carcinoma cell line [41]) DNA in a background of blood DNA isolated from a healthy donor were generated. Specifically, samples 1 to 12 contain 100%, 33.3%, 11.1%, 3.7%, 1.23%, 0.41%, 0.14%, 0.046%, 0.015%, 0.005%, 0.0017%, and 0% of LD583 DNA, respectively (Additional file 2: Table S2). In total, 2 µg of each sample was submitted to Pangea Laboratory and analyzed in triplicates (500 ng each) with Bladder CARE. The number of cancer cells originally used to generate the standards was calculated considering that a single human genomic DNA molecule weighs 3.59 pg [43]. For each sample, standard deviation and standard error were calculated based on the BCI values obtained from each of the three technical replicates. Student’s two-tailed *t* test was performed in order to determine the significance of BCI changes between consecutive samples (Additional file 2: Table S2).

## Supplementary Information

**Additional file 1: Figure S1**. Diagnostic Results of the Logistic Regression Model. Confusion matrix, prediction distributions, and receiver operating characteristic (ROC) curve of the chosen logistic regression model after cross-validation, visualizing the results of the test set (stratified 20% of total data, containing a representative ratio of the control/cancer cohorts). (A) The area under the curve (AUC) of the ROC curve is a measure of model performance when considering model output as a classifier, plotting the difference in true positive rate (sensitivity) and false positive rate (1—specificity) as the classification decision boundary is changed (what threshold of percent probability is required to classify as positive), where 0.5 = no discrimination and 1 = perfect discrimination. (B) The model’s probability outputs for the test set, with the 50% probability decision boundary shown as the dotted line. (C) Confusion Matrix outlining the model classification and true classification of the test set. Mean AUC from stratified *k*-fold cross-validation (*k* = 5) = 0.974 ± 0.02 CI, with mean *F*1 score = 0.886 ± 0.033 CI. Chosen model Log Likelihood Ratio *p* value = 1.047e−36, with pseudo *R*2 = 0.715.

**Additional file 2:** **Table S1.** For each sample the percentage of methylation was calculated by applying the formula 2^-ΔCT * 100, where ΔCT was calculated by subtracting the CT value of the HpaII restriction digestion reaction (HpaII RD) from the CT value of the no restriction digestion reaction (No RD). The test was performed in two technical replicates. **Table S2.** Abbreviations: Rep, replicate; SD, standard deviation; SE, standard error; n.a., not applicable; LD583, cancer cell line [41]. *, number of cells (LD583 cancer cell line) are calculated considering that one DNA molecule weights 3.59 pg [43]. **Table S3.** Abbreviations: TCC, transitional cell carcinoma; n.a., not available. False negative cases are highlighted in grey. Blood and leukocytes contents was measured with Multistix 10SG Urinalysis Test Strips (Siemens), and results are reported based on manufacturer classification.

## Data Availability

The datasets used and/or analyzed during the current study are available from the corresponding author on reasonable request. G. Liang and P. Piatti have full access to all the data in the study and take responsibility for the integrity of the data and the accuracy of the data analysis.

## Abbreviations

BC: Bladder cancer
NMIBC: Non-muscle invasive bladder cancer
MIBC: Muscle invasive bladder cancer
TURBT: Transurethral resection of bladder tumor
MSRE-qPCR: Methylation-sensitive restriction enzyme-qPCR
MS-qPCR: Methylation-specific-qPCR
