# Supplementary figures and images for: Clinical evaluation of Bladder CARE, a new epigenetic test for bladder cancer detection in urine samples
Source: Clin Epigenetics. 2021 Apr 21;13:84. doi: 10.1186/s13148-021-01029-1 (PMC8059345; doi:10.1186/s13148-021-01029-1)

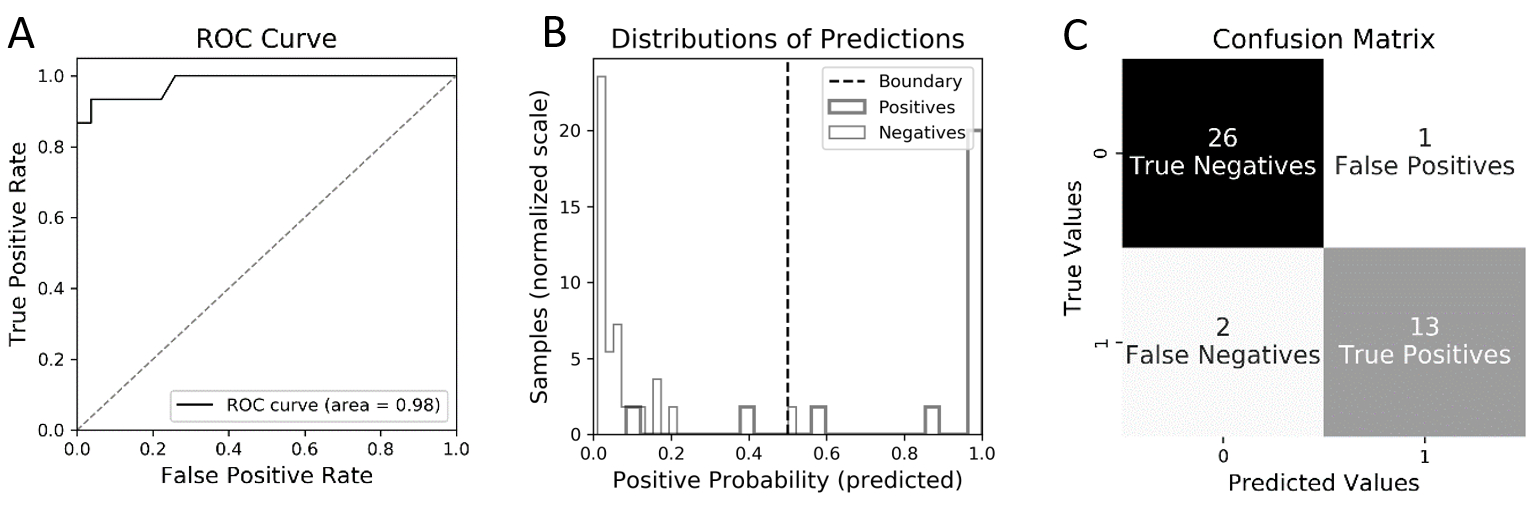

Supplement: Supplementary file 1 — Additional file 1: Figure S1. Diagnostic Results of the Logistic Regression Model. Confusion matrix, prediction distributions, and receiver operating characteristic (ROC) curve of the chosen logistic regression model after cross-validation, visualizing the results of the test set (stratified 20% of total data, containing a representative ratio of the control/cancer cohorts). (A) The area under the curve (AUC) of the ROC curve is a measure of model performance when considering model output as a classifier, plotting the difference in true positive rate (sensitivity) and false positive rate (1—specificity) as the classification decision boundary is changed (what threshold of percent probability is required to classify as positive), where 0.5 = no discrimination and 1 = perfect discrimination. (B) The model’s probability outputs for the test set, with the 50% probability decision boundary shown as the dotted line. (C) Confusion Matrix outlining the model classification and true classification of the test set. Mean AUC from stratified k-fold cross-validation (k = 5) = 0.974 ± 0.02 CI, with mean F1 score = 0.886 ± 0.033 CI. Chosen model Log Likelihood Ratio p value = 1.047e−36, with pseudo R2 = 0.715. [file 13148_2021_1029_MOESM1_ESM.jpg]
