# Supplementary material for: Clinical evaluation of Bladder CARE, a new epigenetic test for bladder cancer detection in urine samples
Source: Clin Epigenetics. 2021 Apr 21;13:84. doi: 10.1186/s13148-021-01029-1 (PMC8059345; doi:10.1186/s13148-021-01029-1)
Supplement: Supplementary file 2 — Additional file 2: Table S1. For each sample the percentage of methylation was calculated by applying the formula 2^-ΔCT * 100, where ΔCT was calculated by subtracting the CT value of the HpaII restriction digestion reaction (HpaII RD) from the CT value of the no restriction digestion reaction (No RD). The test was performed in two technical replicates. Table S2. Abbreviations: Rep, replicate; SD, standard deviation; SE, standard error; n.a., not applicable; LD583, cancer cell line [41]. *, number of cells (LD583 cancer cell line) are calculated considering that one DNA molecule weights 3.59 pg [43]. Table S3. Abbreviations: TCC, transitional cell carcinoma; n.a., not available. False negative cases are highlighted in grey. Blood and leukocytes contents was measured with Multistix 10SG Urinalysis Test Strips (Siemens), and results are reported based on manufacturer classification. [file 13148_2021_1029_MOESM2_ESM.docx]

**SUPPLEMENTARY TABLES**

**Table S1. MGMT methylation in blood DNA and M.SssI methylation efficiency**

|  | No RD | HpaII RD | methylation (%) |
| --- | --- | --- | --- |
| Sample | CT value | CT value |  |
| Untreated blood DNA | 23.26 | 30.83 | 0.53 |
| M.SssI-treated blood DNA | 23.82 | 23.86 | 97.16 |

**Table S1.** For each sample the percentage of methylation was calculated by applying the formula 2^-∆CT * 100, where ∆CT was calculated by subtracting the CT value of the HpaII restriction digestion reaction (HpaII RD) from the CT value of the no restriction digestion reaction (No RD). The test was performed in two technical replicates.

**Table S2. Characteristics and analysis of the spike-in control samples used to calculate Bladder CARE Test LOD and Linearity**

| Sample | LD583 DNA % | Blood DNA % | Cancer cells in 500 ng of DNA* | BCI values | | | Average BCI | SD | SE | p value |
| --- | --- | --- | --- | --- | --- | --- | --- | --- | --- | --- |
|  |  |  |  | Rep 1 | Rep 2 | Rep 3 |  |  |  |  |
| 1 | 100 | 0 | 69638 | 759.0 | 765.8 | 811.7 | 778.8 | 28.68 | 16.56 | 0.00001 |
| 2 | 33.3 | 66.7 | 23213 | 254.2 | 248.8 | 239.4 | 247.5 | 7.48 | 4.32 | 0.00001 |
| 3 | 11.1 | 88.9 | 7738 | 75.7 | 75.3 | 88.1 | 79.7 | 7.31 | 4.22 | 0.00026 |
| 4 | 3.70 | 96.3 | 2579 | 26.6 | 29.0 | 27.1 | 27.6 | 1.28 | 0.74 | 0.00002 |
| 5 | 1.23 | 98.77 | 860 | 9.6 | 9.0 | 10.2 | 9.6 | 0.60 | 0.35 | 0.0001 |
| 6 | 0.41 | 99.59 | 287 | 3.5 | 3.3 | 3.2 | 3.4 | 0.14 | 0.08 | 0.0005 |
| 7 | 0.14 | 99.86 | 96 | 1.5 | 2.0 | 1.7 | 1.7 | 0.23 | 0.14 | 0.036 |
| 8 | 0.046 | 99.954 | 32 | 1.2 | 1.4 | 1.1 | 1.2 | 0.15 | 0.09 | 0.031 |
| 9 | 0.015 | 99.985 | 11 | 0.9 | 0.9 | 1.0 | 0.9 | 0.06 | 0.03 | 0.441 |
| 10 | 0.005 | 99.995 | 4 | 0.7 | 1.0 | 0.8 | 0.8 | 0.12 | 0.07 | 0.338 |
| 11 | 0.0017 | 99.9998 | 1 | 0.8 | 0.7 | 0.8 | 0.8 | 0.03 | 0.02 | 0.648 |
| 12 | 0 | 100 | 0 | 0.8 | 0.8 | 0.7 | 0.8 | 0.08 | 0.04 | n.a. |
| Abbreviations: Rep, replicate; SD, standard deviation; SE, standard error; n.a., not applicable; LD583, cancer cell line [41]. *, number of cells (LD583 cancer cell line) are calculated considering that one DNA molecule weights 3.59 pg [43]. | | | | | | | | | | |
|  | | | | | | | | | | |

**Table S3. Characteristics of the 5 false negative cases in comparison to 16 other randomly chosen cancer samples**

| Sample ID | Tumor Type | Tumor Grade | BCI | Bladder CARE Results | DNA yield (ng/ml urine) | Blood Content | Leukocytes Content |
| --- | --- | --- | --- | --- | --- | --- | --- |
|  |  |  |  |  |  |  |  |
| 02-0007 | TCC | G1 | 1.8 | Negative | 297.8 | Large +++ | Moderate ++ |
| 02-0040 | TCC | n.a. | 1.5 | Negative | 100.2 | Large +++ | Moderate ++ |
| 02-0100 | TCC | G2 | 1.1 | Negative | 58.6 | Large +++ | Moderate ++ |
| 02-0104 | TCC | G2 | 1.4 | Negative | 8.2 | Large +++ | Negative |
| 10/18U | TCC | G2 | 1.6 | Negative | 736.2 | n.a. | n.a. |
| 02-0057 | TCC | G2 | 24.2 | Positive | 65.2 | Large +++ | Moderate ++ |
| 02-0120 | TCC | G2 | 64.4 | Positive | 146.4 | Large +++ | Moderate ++ |
| 5/18U | TCC | G3 | 65.9 | Positive | 295.4 | Large +++ | Moderate ++ |
| 02-0064 | TCC | G1 | 41.7 | Positive | 265.7 | Small + | Moderate ++ |
| 02-0033 | TCC | G3 | 10.4 | Positive | 1281.7 | Small + | Moderate ++ |
| 02-0029 | TCC | G1 | 7.2 | Positive | 391.4 | Large +++ | Small + |
| 02-0123 | TCC | G2 | 265 | Positive | 111.1 | Large +++ | Small + |
| 4/18U | TCC | G2 | 37.7 | Positive | 216.64 | Large +++ | Trace |
| 02-0019 | TCC | G2 | 445 | Positive | 31.9 | Large +++ | Negative |
| 02-0041 | TCC | n.a. | 3.4 | High-Risk | 85.5 | Large +++ | Negative |
| 02-0099 | TCC | G1 | 271 | Positive | 2417.6 | Large +++ | Negative |
| 02-0025 | TCC | G2 | 82.2 | Positive | 5.0 | Hemolyzed Trace | Negative |
| 02-0111 | TCC | G2 | 334 | Positive | 113.6 | Moderate ++ | Negative |
| 02-0118 | TCC | G2 | 21.2 | Positive | 20.3 | Negative | Negative |
| 02-0045 | TCC | G2 | 859 | Positive | 8.3 | Moderate ++ | Negative |

Abbreviations: TCC, transitional cell carcinoma; n.a., not available. False negative cases are highlighted in grey. Blood and leukocytes contents was measured with Multistix 10SG Urinalysis Test Strips (Siemens), and results are reported based on manufacturer classification.
